# Supplementary material for: Insights into the Jasmonate Signaling in Basal Land Plant Revealed by the Multi-Omics Analysis of an Antarctic Moss Pohlia nutans Treated with OPDA
Source: Int J Mol Sci. 2022 Nov 4;23(21):13507. doi: 10.3390/ijms232113507 (PMC9658390; doi:10.3390/ijms232113507)
Supplement: Supplementary file 1 [file ijms-23-13507-s001.zip › Table S3. Summary of data quality in transcriptome sequencing.pdf]

Table S3. Summary of data quality in transcriptome sequencing. Raw reads, the count of original sequence data; Clean reads, the count of the filtered sequencing data; Q20/Q30, the percentages of the bases with phred values larger than 20 and 30 in the total reads obtained by sequencing, respectively.

**Sample**	**Raw Reads**	**Clean Reads**	**Clean Base(G)**	**Error Rate(%)**	**Q20(%)**	**Q30(%)**	**GC Content(%)**
CK_1	46992658	44347034	6.65	0.03	97.96	94.31	52.08
CK_2	46321050	43772700	6.57	0.03	97.85	94.05	52.07
CK_3	45777924	43642126	6.55	0.03	97.75	93.66	52.47
OPDA_1	45554002	42164180	6.32	0.03	97.88	94.08	52.11
OPDA_2	45664516	41413196	6.21	0.03	97.97	94.13	51.89
OPDA_3	44301520	40420748	6.06	0.03	97.98	94.15	52.1
