# Supplementary material for: Insights into the Jasmonate Signaling in Basal Land Plant Revealed by the Multi-Omics Analysis of an Antarctic Moss Pohlia nutans Treated with OPDA
Source: Int J Mol Sci. 2022 Nov 4;23(21):13507. doi: 10.3390/ijms232113507 (PMC9658390; doi:10.3390/ijms232113507)
Supplement: Supplementary file 1 [file ijms-23-13507-s001.zip › Table S6. Primers used for quantitative real-time RT-PCR analysis.pdf]

Table S6. Primers used for quantitative real-time RT-PCR analysis.

Gene ID	Gene symbol	Primer name	Primer sequence (5'-3')	Annealing temperature (°C)	Product length (bp)
Poh0285230.1	AOS-01	AOS-01F	CACTACTCAAGCAAGCACGGACAT	59.9	152
		AOS-01R	CGCTGAATCGGTGGGTCAATGG	60.2	
Poh0370410.1	AOC-01	AOC-01F	GCACAGTCTTCCGCACCAACAT	60	176
		AOC-01R	CATCTCCATTCCTCCCGTGTACGA	60.3	
Poh0051570.1	OPR-01	OPR-01F	TCGGGAGGAAATGGTAGCAATGGT	60.4	171
		OPR-01R	ATCATGTCGGTAGCCAGCGTGTT	60.9	
Poh0172290.1	PYL-01	PYL-01F	GGAGCTATGGCATTGACAGGACTG	59.8	183
		PYL-01R	TGAGAGAGTGAGCGAAGGGTTGTAT	59.9	
Poh0036960.1	PP2C-01	PP2C-01F	GTAAGTTGCGAAGGAGCGTGGTT	60.1	177
		PP2C-01R	GACAGAACGAACGGCAAGTTGAAC	59.6	
Poh0360030.1	SnRK2-01	SnRK2-01F	GACGATGTAACTGTGGAGTGCTGTG	60.5	165
		SnRK2-01R	CCTCGGACGCAATATACACCTACG	59.5	
Poh0016430.1	SnRK2-02	SnRK2-02F	GCCGCTCGCCAACATCAACAT	60.4	157
		SnRK2-02R	CGCAACAGCAAGGAGAGGACTC	59.6	
Poh0324020.1	ABI5-02	ABI5-02F	CGAGCGTGGTGTTGTATTGTAGGT	59.7	155
		ABI5-02R	GTGTGGCGAAGTGAATGGCTGTAT	60.2	
Poh0024230.1	JAZ-01	JAZ-01F	CTGCTCCTGACCGTGACTCGTA	59.8	148
		JAZ-01R	TGGTTGGAAGGGTTGTGCTCTG	59	
Poh0281560.1	JAZ-02	JAZ-02F	TCTCCAACATTCGACCACCACC	58.6	130
		JAZ-02R	GCTTCGCCTTCCTCACTCACA	58.5	
Poh0084220.1	CHO1-02	CHO1-02F	GCTTCGTTCTTGATGTGTTGATTTGC	59.2	144
		CHO1-02R	CCAGCGTTCTCCTTACCTTACCAT	58.7	
Poh0018780.1	PISD-02	PISD-02F	CAGGTCACTTCGGCACAGATGG	59.6	141
		PISD-02R	GTCCTCCAACACCATGCTTCTCAC	60.2	
Poh0120240.1	PLA2G-01	PLA2G-01F	CAACGACAATCAACCGCCATAGC	59.2	152
		PLA2G-01R	GTGACCGACCACCACCATCATC	59.3	
Poh0050830.1	GDE1-01	GDE1-01F	ATTGCGGTATAGGCTACACAGGTTG	59.6	165
		GDE1-01R	TCGCCGTCTTGAACTGCTGAAC	59.8	
Poh0219590.1	GDE1-02	GDE1-02F	GTATAGGCTACACAGGTTGCGATGG	59.9	159
		GDE1-02R	TCGCCGCCTTGAACTGCTGAAC	62.2	
Poh0323340.1	EPT1-01	EPT1-01F	CTTCACAGACACGCTCATCCTTCC	60	182
		EPT1-01R	CGAACACCAGCACAAGAGACACAT	60.1	
Poh0307150.1	PEMT-02	PEMT-02F	GGTCATTGAGCGAGAAGAAGATCCG	60.2	170
		PEMT-02R	CACCCGAATCAGTGAAGAACATTGC	59.6	
Poh0348200.1	CPT1-01	CPT1-01F	CTTCACAGACACGCTCATCCTTCC	60	182
		CPT1-01R	CGAACACCAGCACAAGAGACACAT	60.1	
Poh0234360.1	CHS-01	CHS-01F	CTGAGCCTGACGACTGACAAGATG	59.8	172
		CHS-01R	CGAAGCCGATGAAGAATCCGAACT	59.8	
Poh0239650.1	CHS-02	CHS-02F	GCTGGAGTATGGCAAGTGGAAATCT	59.8	150
		CHS-02R	TGTCTGTCTGTCTGTCTGTCTGTCT	59.5	

Poh0039230.1	CHI-01	CHI-01F	CCATCGCCATCGCCAGTTGTT	59.8	150
		CHI-01R	AGCTGCCAGGAGGAGTAAGAGAC	59.6	
Poh0178020.1	CHI-02	CHI-02F	ACAGTTGGTGCTTGTACGAGACATC	59.9	172
		CHI-02R	TGTGATAGTGGTGCCATTGCTGAG	59.7	
Poh0205200.1	F3'H-01	F3'H-01F	GGCGTCATCTCCGAACCATCTG	59.4	168
		F3'H-01R	TGGTAGCCGTGTTCCGCAACT	60.5	
Poh0004720.1	F3'H-02	F3'H-02F	CTTCGCCTCGTATGCCGTGTTC	60.3	172
		F3'H-02R	CGTCATGTTCTTCCTCCGTTCCTC	59.7	
Poh0056150.1	2-OGD-01	2-OGD-01F	CCCTGCGGTTGTTTCGGTTGAT	60	191
		2-OGD-01R	GGTGAACAGATTGGCGTCATGGT	59.8	
Poh0225840.1	2-OGD-02	2-OGD-02F	CGAGGAGCAGCAGGAGTTGTTC	59.6	200
		2-OGD-02R	GACACGGCGGGTAATGGTTCAAC	60.7	
Poh0251360.1	DFR-03	DFR-02F	ATGTGCTTGGTCCGAGTGTGATTG	60.2	169
		DFR-02R	TCTCCACGACGCTTCTCCTTCTC	60.1	
Poh0007930.1	DFR-04	DFR-03F	TGCTGGTCTGAAGGCTGTAGGC	60.6	169
		DFR-03R	ACAAGGTACGGTCTCTGGTTGGC	60.8	
Poh0204970.1	PnGAPDH	PnGAPDH-1q5	AGGAAGGACTCGCCTCTGGAAG	59.5	142
		PnGAPDH-1q3	CGATACTGATGCCGTCGTTGCC	60.3	
Poh0314480.1	Actin-1	PnAct1-qPCR1	CGGAAACATCGTGCTGAGTGGAG	60.2	185
		PnAct1-qPCR2	ACATCTGCTGGAAGGTGCTGAGA	60	
Poh0012540.1	β-tubulin-1	PnTub1-qPCR1	TCGTCGTACTTCGTGGAGTGGA	59.2	160
		PnTub1-qPCR2	TCCTGAACATCGCCGTGAACTG	59.2	
